# Characterization of Amorphous Solid Dispersion of Pharmaceutical Compound with pH-Dependent Solubility Prepared by Continuous-Spray Granulator

**DOI:** 10.3390/pharmaceutics11040159

**Published:** 2019-04-03

**Authors:** Ryoma Tanaka, Yusuke Hattori, Yukun Horie, Hitoshi Kamada, Takuya Nagato, Makoto Otsuka

**Affiliations:** 1 Graduate School of Pharmaceutical Sciences, Musashino University, 1-1-20 Shin-machi, Nishi-Tokyo, Tokyo 202-8585, Japan; g1878005@stu.musashino-u.ac.jp (R.T.); yhattori@musashino-u.ac.jp (Y.H.); 2 Department of Pharmaceutics, College of Pharmacy, University of Minnesota, Minneapolis, MN 55455, USA; 3 Faculty of Pharmacy, Musashino University, 1-1-20 Shin-machi, Nishi-Tokyo, Tokyo 202-8585, Japan; s1343033@stu.musashino-u.ac.jp; 4 Research Institute of Pharmaceutical Sciences, Musashino University, 1-1-20 Shin-machi, Nishi-Tokyo, Tokyo 202-8585, Japan; 5 Research & Development Department, Technical Division, Powrex Corporation, 5-5-5 Kitagawara, Itami, Hyogo 664-0837, Japan; kamada@powrex.co.jp (H.K.); t-nagato@powrex.co.jp (T.N.)

**Keywords:** amorphous, solid dispersion, molecular complex, rebamipide, polymer, interaction, stability, characterization, continuous processing, granulation, process development

## Abstract

A continuous-spray granulator (CTS-SGR) is a one-step granulation technology capable of using solutions or suspensions. The present research objectives were, (1) to reduce the manufacturing operations for solid dosage formulations, (2) to make amorphous solid dispersion (ASD) granules without pre-preparation of amorphous solids of active pharmaceutical ingredients (API), and (3) to characterize the obtained SGR granules by comprehensive pharmaceutical analysis. Rebamipide (RBM), a biopharmaceutical classification system class IV drug, that has low solubility or permeability in the stomach, was selected as a model compound. Five kind of granules with different concentrations of polyvinylpyrrolidone/vinyl acetate copolymer (PVP-VA) were prepared using a one-step SGR process. All of the SGR granules could be produced in amorphous or ASD form and their thermodynamic stability was very high because of high glass transition temperatures (>178 °C). They were unstable in 20 °C/75%RH; however, their stability was improved according to the proportion of polymer. The carboxy group of RBM was ionized in the granules and interactions appeared between RBM and PVP-VA, with the formation of an ASD confirmed and the solubility was enhanced compared with bulk RBM crystals. The SGR methodology has the possibility of contributing to process development in the pharmaceutical industry.

## 1. Introduction

Among recently developed pharmaceuticals, many synthesized candidate drug compounds have a low bioavailability, due to their low aqueous solubility and/or permeability [1]. These issues influence discovery stage studies and lead to delays in the development of new drugs. According to the biopharmaceutical classification system (BCS), the drug dissolution profile and solubility of the solid-state form are major factors and they influence gastrointestinal permeability, bioavailability, and clinical response [2]. Hence, it is necessary to improve bioavailability by enhancing the solubility of poorly water-soluble drug compounds by making the most of pharmaceutical technology [3,4]. The main methodologies reported to achieve this include alteration of the solid-state by amorphization [5], increasing the particle surface area by size reduction [6], and the formation of pharmaceutical molecular complexes [7,8].

Among these, amorphous solid dispersion (ASD) is a representative of amorphous molecular complexes [9,10]. Generally, ASD is a solid of a polymer-based material involving a homogeneously dispersed active pharmaceutical ingredient (API) molecules in a disordered state. In some cases, a complex between more than two APIs is dispersed in a polymer material [11,12]. The amorphous state of a compound is more unstable without long-range order, compared with the crystal state [13]. The use of amorphization or disruption of the crystal lattice increases the solubility and, consequently, leads to improved bioavailability. As an advantage of the ASD system, the presence of a polymer can stabilize an amorphous API. Some studies on ASDs have investigated API-polymer interactions and relationships [14,15], manufacturing process development [16,17], and other factors [18,19,20]. The preparation of an ASD can be chosen dependent on the nature of the drug, for example, the melting method [21,22] and the solvent method [23,24]. These preparations are necessary to provide the final product of ASD formulations, but the manufacturing unit operation cutbacks are ideal from the viewpoint of high-level quality control and cost saving in the recent pharmaceutical industry. In addition, an accomplishment for high drug loading granules or tablets is difficult with ASD formulations because of the necessity for high concentrations, such as 70−80%, of polymer carriers to enhance the solubility, stability, and drug-polymer interaction [25,26]. Polymers have been used extensively to fabricate ASDs; however, the formation of stable ASDs requires high polymer concentrations, limiting their use with low-dose APIs. Therefore, considering an alternative methodology for solid dosage formulations, ASD granules with high drug loading, using restricted manufacturing processes, may be suitable.

**Figure 1:**
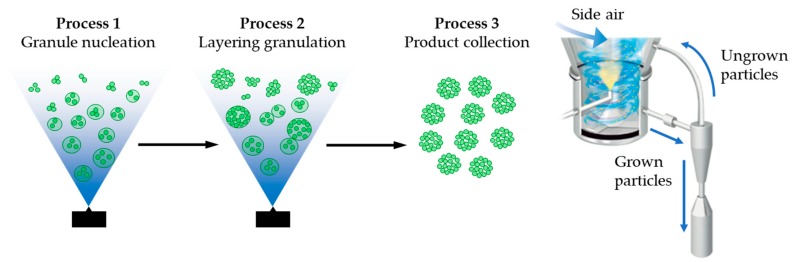
The circulation system of a continuous-spray granulator (CTS-SGR).

A continuous-spray granulator (CTS-SGR) provides a one-step granulation method from solution or suspension. The SGR method can be divided into the following three processes (Figure 1): Granule nucleation by spray drying, layering granulation by continuous spray, and product collection using a size classification system. A two-fluid spray nozzle is placed vertically at the bottom. The SGR also has side air nozzles, which act to brush the adhered powder off the face of the wall, to maintain a continuous flowable state in the SGR. The SGR system resembles an ordinary spray drier and fluid bed granulation, which together result in layering granulation [27,28]. The granule size of spray drying and fluid bed granulation depends on the particle size in the suspension sample, the nozzle orifice diameter, and the airflow. However, spray drying granulation may have technical problems, such as the homogeneity of the product [29]. Additionally, layering granulation requires core particles as a seed for the granule in order to obtain the comparatively large size granules in general. Thus, the formation a granule with high drug loading is difficult because of the concentration of the seed in the granule [30]. On the other hand, SGR provides enhanced handling, such as the flowability of bulk powder by layering granulation without core particles, and can prepare uniformly spherical granules until the desired granule size and shape is reached.

Rebamipide (RBM), a gastroprotective agent prescribed for gastric ulcer and gastritis patients, is a weakly acidic BCS class IV drug that is insoluble in acid conditions. However, dissolution in a low pH environment is needed because the precise mechanisms of RBM involve increasing gastric mucosal prostaglandin and gastric mucus production and the site of action of RBM is the stomach [31]. Commercial RBM tablets contain 100 mg of the API and the prescribed dosage is three times per day. The current study aimed to combine a high API loading formulation and enhancement of the solubility of RBM by specific manufacturing processes. For the purpose of achieving this, ASD granules were prepared using SGR as a one-step method for producing high drug loading granules. Five types of granules with different concentrations of polymers (0%, 5%, 10%, 20%, and 30% as weight ratio) were prepared by SGR. The physical properties, stability, thermal behavior, molecular state, and solubility of the obtained SGR samples were investigated. Comprehensive identification was performed to understand the characteristics of the granules and expand the possibility of SGR as a process development.

## 2. Materials and Methods

### 2.1. Materials

**Figure 2:**
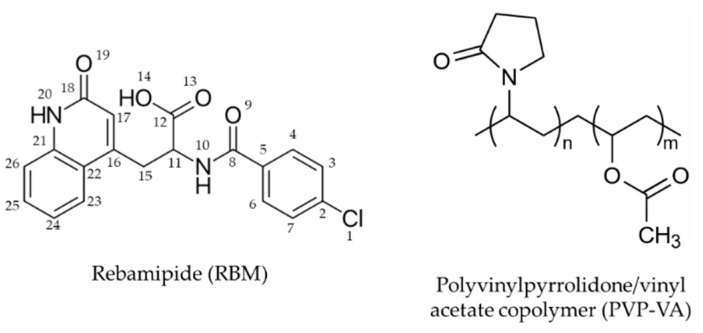
Chemical structures of rebamipide with atom numbering and polyvinylpyrrolidone/vinyl acetate copolymer (Kollidon^®^ VA64).

RBM of pharmaceutical grade was kindly provided by Ohara Pharmaceutical (Shiga, Japan, Figure 2). Sodium hydroxide (NaOH) as an alkylating agent was purchased from Fujifilm Wako Pure Chemical (Osaka, Japan). Polyvinylpyrrolidone/vinyl acetate copolymer (PVP-VA; Kollidon^®^ VA64, Figure 2) as a carrier was a generous gift from BASF (Ludwigshafen, Germany). As the additives for the tablets, magnesium aluminometa silicates (MAS; Neusilin^®^ NS2N) was a gift from Fuji Chemical Industry (Wakayama, Japan) and croscarmellose sodium (CCS; Kiccolate^TM^) and magnesium stearate (Mg-St) were purchased from Asahi Kasei Chemicals (Tokyo, Japan) and Fujifilm Wako Pure Chemical, respectively. All other chemicals were commercially available products of analytical grade.

### 2.2. Granule Preparation Using a Continuous-Spray Granulator

Table 1 shows the summarized liquid formulation for spraying using SGR. Firstly, RBM, PVP-VA, and NaOH totaling 200 g were dissolved in 1800 g of purified water at 80 °C, with stirring. At this time, the molar ratio of REB and NaOH was fixed at 1:1 and PVP-VA in Runs 1−5 accounted for 0%, 5%, 10%, 20%, and 30% as the weight ratio. Then, the sample solutions were fed into the SGR (CST-SGR-01 without size classification system; Powrex, Hyogo, Japan) at a rate of 10−15 mg/min and sprayed using a two-fluid nozzle with the following conditions: Atomizing air rate was 40–80 NL/min, inlet air temperature was 75 °C, and running time was 120–150 min.

### 2.3. Physical Property Measurements

A scanning electron microscope (SEM; JSM-6510LV, JEOL, Tokyo, Japan) was used to characterize the particle state and morphology. Granule samples were sprinkled onto a carbon tape and coated with carbon by a JEC-560 (JEOL). The acceleration voltage, magnification, and working distance were 1.0 kV, ×500, and 8 mm, respectively.

Dried particle size distribution was investigated using a laser light scattering particle analyzer (Mastersizer 3000E with Aero M, Malvern Panalytical, Malvern, UK). Data analysis was done based on algorithms utilizing Mie scattering theory for non-spherical materials. The results were represented as mass median diameter (D50) with a standard deviation of (n=5).

The angle of repose (AR) was evaluated using a modified tilting method [32]. Approximately 30 mg of the granule sample was fed into a sample holder. Then, the holder was slowly tilted until the sample began to slide and the angle of the tilt was measured. The results were described as the mean angle of repose with a standard deviation of (n=30).

The bulk density 1 ($\rho_{B}$) was measured by filling a graduated cylinder (50 mL) with a certain amount of each sample, the height of sample was approximately 10 mm. Additionally, the tapped density ($\rho_{T}$) was evaluated by tapping down each sample in the cylinder, the tapping was repeated 70 times, and the value of the Hausner ratio (HR) and Carr index (CI) were calculated using the following equations:

(1) $$HR=\frac{\rho_{T}}{\rho_{B}}$$

(2) $$CI\left(\%\right)=100\left(1-\frac{\rho_{B}}{\rho_{T}}\right)$$

where B and T are bulk and tapped samples, respectively [33,34]. The results were shown as mean values with a standard deviation of (n=3).

### 2.4. Stability Testing at Different Humidities by X-Ray Diffractometry

In order to compare the stability of the amorphous state of RBM in each granule type, 4 g of each sample was placed into containers at 30% RH or 75% RH, with an ambient temperature ca. 20 °C, for the specified time periods (6 months at maximum). The samples were promptly measured by X-ray diffraction (XRD) upon removal of the lid at each time point.

The XRD pattern of each sample was collected using RINT-Ultima III (Rigaku, Tokyo, Japan) with Cu Kα radiation (40 kV × 40 mA). The diffraction angle range was from 5° to 45° in 2-theta, with a step of 0.02° and scanned at 15°/min. Relatively large granules were ground using manual grinding in an agate mortar for adequate XRD analysis.

### 2.5. Thermal Analysis

A differential scanning calorimeter (DSC7000X, Hitachi, Tokyo, Japan) was used for investigating the thermal behavior of granule samples. An approximately 5 mg sample was placed in an aluminum DSC pan. All the measurements were done under a dry nitrogen purge at 30 mL/min and heated from 25 °C to 350 °C at a rate of 5 °C/min. For the purpose of identification of the glass transition temperature (*T*_g_), DSC was operated in the modulated mode with the following conditions: Temperature modulation was ±3 °C, the repetition rate was 0.2 Hz, and the heating rate was 5 °C/min. The value of the glass transition of a binary system was predicted using the following Couchman−Karasz equation:

(3) $$T_{g\;calc}\left({}^{\circ}C\right)=\frac{w_{1}T_{g1}+Kw_{2}T_{g2}}{w_{1}+Kw_{2}}$$

(4) $$K=\frac{\Delta C_{p2}}{\Delta C_{p1}}$$

where $T_{g\;calc}$ is the theoretical glass transition (°C), $w_{1}$ and $w_{2}$ are the weight fractions of each component, $T_{g1}$ and $T_{g2}$ are their glass transitions, and $\Delta C_{p1}$ and $\Delta C_{p2}$ are the change in specific heat capacity at the glass transition [35,36]. Additionally, a positive difference between the measured and calculated glass transition temperatures was obtained as the characteristic parameter of interaction.

### 2.6. Fourier Transformed Infrared Spectroscopy

Infrared (IR) spectra were accumulated using a Fourier-transform IR spectrometer (FT/IR-4100, Jasco, Tokyo, Japan). The spectral data were collected by powder diffuse reflectance using KBr powder, with 64 scans at 8 cm^−1^ resolution.

### 2.7. Tablet Preparation and Dissolution Testing

A mixture of resulting granules with specific amounts of PVP-VA, 7.2 mg of MAS (3.6%), and 10.0 mg of CCS (5.0%) was blended with 1.0 mg of Mg-St (0.5%) just before tableting. Each formulation ratio of RBM per tablet was adjusted to the same amount as commercial Mucosta^®^ (114.2 mg as a total API). Additionally, the PVP-VA amount depended on each batch (54.5 mg as total PVP-VA). Specifically, the amounts in batch No. 1−5 granules and additional PVP-VAs in tablets were 127.2 mg (63.6%) and 54.6 mg (27.3%), 136.4 mg (68.2%) and 45.4 mg (22.7%), 145.4 mg (72.7%) and 36.4 mg (18.2%), 163.6 mg (81.8%) and 18.2 mg (9.1%), and 181.8 mg (90.9%) and 0 mg (0.0%), respectively. The mixture was compressed using 8 mm flat-faced punches in a single stroke tablet press (Handtab-100, Ichihashi Seiki, Kyoto, Japan). The tablet weight and hardness were 200 mg and 40 N (60–80 MPa compression pressure), respectively.

Dissolution testing of tablets was carried out in 900 mL water as the test medium (37.5 ± 0.5 °C) using an NTR-3000 apparatus with a paddle speed of 50 rpm (Toyama Sangyo, Osaka, Japan) and a S-2450 spectrophotometer (Shimadzu, Kyoto, Japan). In addition, granule dissolution testing was demonstrated in 900 mL of acidic aqueous solution (pH 1.2 buffer; 37.5 ± 0.5 °C) using a DT-610 apparatus with 100 rpm (Jasco) and a V-530 spectrophotometer (Jasco) because of the low solubility of RBM in an acidic medium. The concentration of API during these tests was determined using a UV/VIS spectrophotometer at 327 nm. The mean values and standard deviations with time were calculated (*n* = 3).

## 3. Results and Discussion

### 3.1. Morphology and Physical Properties

**Figure 3:**
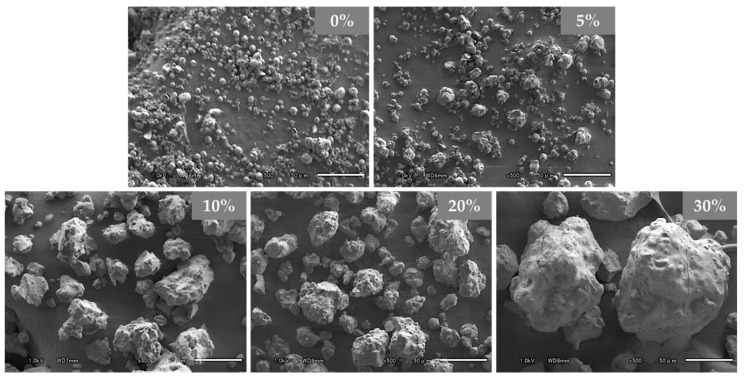
SEM images of the 0−30% PVP-VA granules at the same magnification (×500). The scale bar indicates 50 μm.

RBM granules, in a dry state, were prepared by SGR. Figure 3 shows SEM images of the obtained samples, which provided insights into the morphology and approximate particle size. The particle size of the 0% PVP-VA sample was the smallest and formed microspheres similar to a spray dried sample, whose size would become the nuclei of the granule. Proportionally to the concentration of PVP-VA, the size of granules increased and the surface became smoother because of the high concentration of polymeric carrier presence as a binder. The 30% PVP-VA sample appeared as heavy and dense granules and the surface rarely had pores, because layering granulation was carried out by continuous spraying of the polymer. In addition, the repeating side airs broke the surface roughness of granules and the spray fluid was extended at the granule surface with drying. Therefore, granule spheroidizing was performed. Table 2 shows a summary of the physical properties of the samples. The bulk density ($\rho_{B}$) and tapped density ($\rho_{T}$) increased with layering and the flowability was also enhanced. These prepared granules tended to become denser with SGR and a drastic change in density was observed at >10% concentration in the polymer granules. The granule particle size, size distribution, and particle configuration can be controlled by the concentration of the polymeric binder and the processing time of SGR.

### 3.2. Stability of Amorphous Solid Dispersion

**Figure 4:**
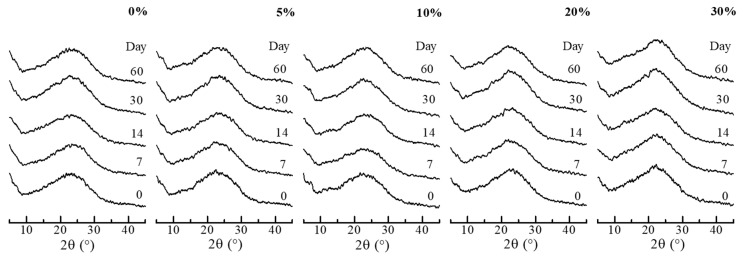
XRD patterns of SGR granules after storage at 20 °C/30% RH. Percentages indicate the weight percentage of PVP-VA in the granules.

**Figure 5:**
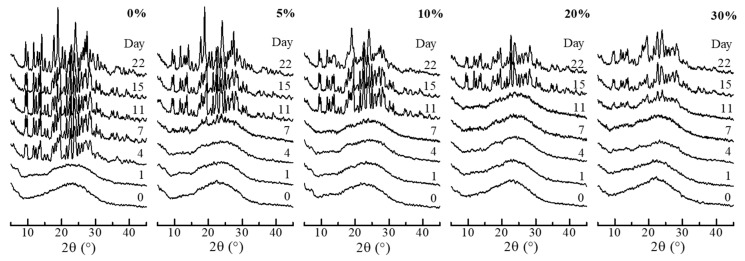
XRD patterns of SGR granules after storage at 20 °C/75% RH. Percentages indicate the weight percentage of PVP-VA in the granules.

In efforts to gain an insight into the crystallization properties, amorphous state stability testing was performed in conditions of high and low humidity and the thermal analysis investigated the thermodynamic and phase behavior. The SGR granules had been confirmed to have no moisture content, such as free and crystal water, by thermal analysis, then the stability testing was carried out. Figure 4 and Figure 5 show the compiled XRD patterns of storage at the conditions of 20 °C/30% RH and 20 °C/75% RH, respectively. Each XRD pattern on the first day (0 day) was a diffraction halo, indicating RBM in granule was an amorphous state in the polymeric matrix by SGR, despite the various PVP-VA concentrations. The amorphous state was maintained for 6 months at 20 °C/30% RH. Under the high relative humidity conditions (20 °C/75% RH), stability was improved depending on the concentration of PVP-VA. The samples with 0%, 5%, 10%, 20%, and 30% PVP-VA crystallized after 4, 7, 11, and 15 days. Relatively stable ASD granules could be prepared with a high concentration of PVP-VA. According to Hancock [36], the glass transition temperature of polymer decreases with the increase in water content in the polymer matrix. Hence, the glass transition temperature of amorphous RBM may become lower and the amorphous state is destabilized due to compositing with the polymer. However, the stability of the amorphous state was improved with increasing the polymer content. It suggests that the interaction between RBM and PVP-VA restricts crystallization of RBM. Notably, the diffraction peaks of granules, after crystallization, was different from RBM, as is. There is a possibility that REB formed co-amorphously with sodium ions in the SGR granule and a sodium salt of RBM was formed or crystallized by absorption of moisture. The interaction between RBM and sodium ions is discussed in FT-IR analysis part.

**Figure 6:**
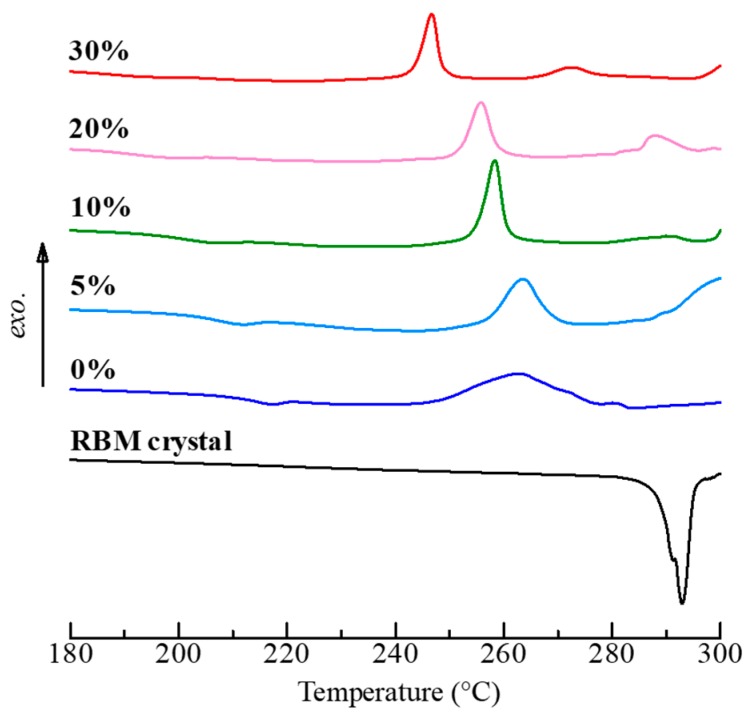
Total heat flow curves of RBM crystals and SGR granules with 0−30% PVP-VA by DSC.

Figure 6 shows DSC curves of the SGR granules. Each granule had a baseline shift, according to the glass transition, at approximately 200.0 °C and the crystallization temperatures of 0%, 5%, 10%, 20%, and 30% PVP-VA granules were 263.9 °C, 263.5 °C, 258.6 °C, 255.7 °C, and 246.7 °C, respectively. Hence, it was confirmed that the samples were established as ASD granules. To analyze the glass transition in detail, modulated DSC was employed, and the results are shown in Table 3. Glass transition temperature is well-known as an important value of ASD. The observed glass transition of the 0% PVP-VA granule (amorphous RBM) was 215.4 °C, while unprocessed pure PVP-VA was 107.8 °C, which meant amorphous RBM originally has a high thermodynamic stability. The reason why the glass transition temperature decreased with increasing concentrations of the polymer was due to the addition of PVP-VA to the formulation. Furthermore, the value of Δ*T*_g_ represent a specific interaction between the API and the polymeric carrier, because the positive deviation reflects an increase in interactions. The deviation appeared when the number and strength of interactions between homo-materials were lower than between hetero-materials [35,36,37]. The calculated value showed that SGR granules with PVP-VA have interactions that lead to time scale stabilization, as with the results of XRD. The strength increased with the PVP-VA concentration. On the other hand, the 0% PVP-VA granule interaction was low because of the absence of the polymer. These results of XRD and DSC demonstrated that the thermodynamic stability of the ASD granules by SGR was sufficiently high because the glass transition was over 178.7 °C and also, even pure PVP-VA was high enough (107.8 °C), whereas undesirable crystallization was provoked under the high humidity condition (20 °C/75% RH). The effect of humidity on the crystallization can be prevented by using a high concentration of polymer or granule coating [38,39].

### 3.3. Molecular State

**Figure 7:**
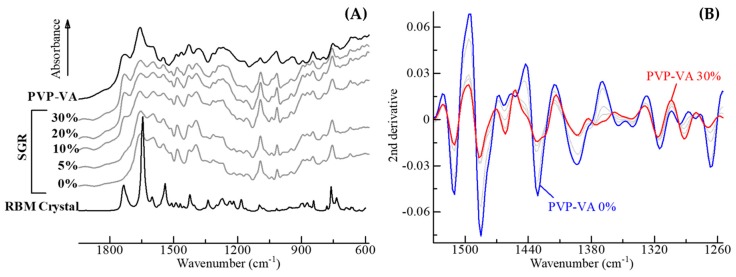
(**A**) Overlays of IR spectra of RBM crystals alone, PVP-VA alone, and SGR granules including 0−30% PVP-VA. (**B**) Representation of the second derivatives of SGR granules.

SGR granules have intermolecular interactions between the API and PVP-VA in proportion to the polymer concentration, as described in the preceding section. This section explains in detail the molecular state change of the granules, according to the interaction. Figure 7A shows the IR spectra of ASD granules by SGR. Bulk RBM crystals had an absorption peak at 1735 cm^−1^, due to the C12*=*O13 stretching vibration. However, this peak disappeared after the SGR process and different peaks at 1595 cm^−1^ and 1394 cm^−1^, which correspond to COO^−^ asymmetric and symmetric stretching vibrations, appeared. These indicated that ionization had occurred due to deprotonation at the carboxy group. Hence, the carboxylate COO^−^ group is suggested to exist without ionic interaction with sodium ions. The characteristic peak of RBM crystals at 1645 cm^−1^, which was assigned to the amide I band of C8=O9 and C18=O19, disappeared in the granules. Bulk PVP-VA peaks of C=O stretching vibration in ester and the amide I band were 1724 cm^−1^ and 1655 cm^−1^, respectively. The other peaks of the amide II and III bands were weak, as shown in Figure 7A.

To detect some weak peaks, second derivative spectra of the SGR granules were calculated (Figure 7B). The second derivative preprocessing allows more specific identification of weak peaks in the original spectra and offers the cancelling of baseline shift [40]. The positive value of the original data was converted into a strong negative peak using the second derivative. The characteristic peaks of RBM at 1480 cm^−1^, 1510 cm^−1^, and 1540 cm^−1^ indicated C8-N10 coupled with N10-H and C18-N20 coupled with N20–H vibrations (amide II band). These bands, which were based on amides, appeared strongly in granules, because the interaction between molecules of RBMs may be weakened by the amorphization process. These bands were also shifted or disappeared in proportion to the PVP-VA concentration, the amide of RBM interacts with the matrix though hydrogen bonding. Therefore, the interaction between RBM and PVP-VA was present in SGR granules. The indication corresponded to the result of Δ*T*_g_ analysis, shown in Table 3. 

### 3.4. Dissolution Ability

**Figure 8:**
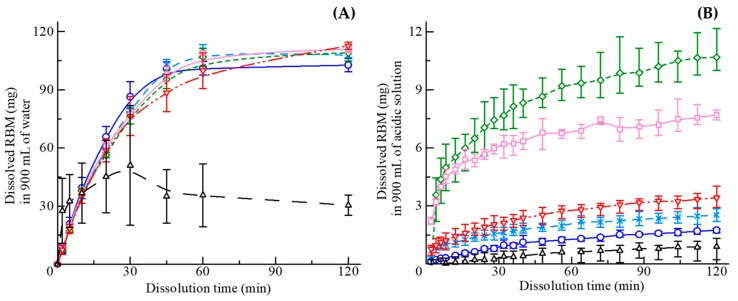
Integrated dissolution profiles in 900 mL of (**A**) tablets in water and (**B**) granules in an acidic solution of RBM crystal (dark triangles), PVP-VA 0% (blue circles), PVP-VA 5% (light blue crosses), PVP-VA 10% (green diamonds), PVP-VA 20% (light red rectangles), and PVP-VA 30% (red inversed triangles). Percentages denote the concentration of PVP-VA in the SGR granules.

Figure 8A shows the dissolution profiles of the tablets in water. All tablets, including SGR granules, showed enhanced solubility of the RBM crystals and the dissolved concentration reached almost 100% (126.67 μg/mL) at 120 min, whereas RBM crystals dissolved slowly. In Figure 8B, dissolution testing using an acidic medium was performed with dispersible SGR granules, due to the low solubility of weakly acidic RBM. The dissolution profiles in the acidic solution were different compared with in water. All of the SGR granules were more soluble than RBM crystals; however, the profiles depended on the concentration of PVP-VA in the granules. In low PVP-VA formulations, such as 0% and 5%, the dissolved concentration reached approximately 2.22 μg/mL. Granules with 10% PVP-VA reached the highest solubility (11.89 μg/mL) among the samples shown in Figure 8B, which was about 10 times higher than RBM crystals. In granules, including 20% and 30% PVP-VA, gelation was observed and the dissolved amount of RBM, at 120 min, decreased with increasing concentration of the polymer. Generally, the recrystallization and crystal growth were prevented by the interaction with the polymer; thus, the polymer contributes to further improvement of the API solubility [41,42]. However, the wettability and swellability of granules were in proportion to the concentration of polymer, while the dispersibility of granules was decreased. Higher the content rate of the polymer caused aggregation and gelation in the vessel, consequently leading to lower drug release from granules in this study. It should be noted that the dissolution profile may change if the amount of contents was revised. Optimized formulation ratios, depending on respective objectives, are vital for the pharmaceutical industry.

The drug release kinetics from ASDs were explained based on mathematical modeling analysis [43,44,45]. As the result of an *R^2^* analysis of least-squares fitting, the best-fitted was the Korsmeyer−Peppas model. The model is generally resumed to the following expression:

(5) $$\frac{M_{t}}{M_{\infty }}=kt^{n}$$

where $M_{t}/M_{\infty }$ is the amount of drug released on time t per unit area, k is the kinetics constant, and n is the release exponent [43,44]. Each *R*^2^ value of the RBM crystal and the granules including 0−30% PVP-VA in acidic medium was 0.917, 0.989, 0.996, 0.987, 0.930, and 0.997. Additionally, each exponent n value was 1.16, 0.64, 0.34, 0.31, 0.26, and 0.38, respectively. The exponents indicate the diffusional drug release mechanism from a matrix, n < 0.43, 0.43 < n < 0.85, and 0.85 < n describes Fickian diffusion, anomalous (non-Fickian) transport, and super case II transport, respectively [43,44]. These results suggested the release mechanisms of the enclosed REB, during the dissolution, were changed into Fickian diffusion due to forming ASD by PVP-VA. The release kinetics from the tablet in water was also explained by the Korsmeyer−Peppas model. Hence, the drug release mechanism after the disintegration of the tablet was the same as the granule.

## 4. Conclusions

One-step granulation methods such as CTS-SGR could be used to prepare ASD granules with high RBM loading to enhance the solubility of this BCS class IV drug. This method involves no specific preparation and produces the ASD granules using a continuous spraying and layering system. According to the concentration of PVP-VA, the obtained granules were found to be heavy and dense, with a smooth surface. The thermodynamic stability of SGR granules was relatively high and the humidity stability at 20 °C/75% RH depended on the concentration of PVP-VA. Molecular interactions formed between RBM and PVP-VA, including the carboxy group of RBM becoming ionized in SGR granules. Dissolution testing demonstrated the improved water solubility of RBM, even in acidic media due to the formation of an ASD. The SGR method, which directly generates granules from solutions, has the possibility to reduce manufacturing operations. The SGR methodology can contribute to new process development in the pharmaceutical industry.

## Figures and Tables

**Table 1:** Materials used for preparing granules using SGR.

Batch No.	RBM ^1^ (g)	PVP-VA ^2^ (g)	NaOH ^3^ (g)	H_2_O ^4^ (g)	Total (g)
1	179.65	0	20.35	1800	2000
2	170.67	10	19.33	1800	2000
3	161.68	20	18.32	1800	2000
4	143.72	40	16.28	1800	2000
5	125.75	60	14.25	1800	2000

^1^ Rebamipide, ^2^ Polyvinylpyrrolidone/vinyl acetate copolymer, ^3^ Sodium hydroxide, ^4^ Water.

**Table 2:** Physical properties of SGR granules.

PVP-VA ^1^ (%)	*ρ*_B_^2^ (g/cm^3^)	*ρ*_T_^3^ (g/cm^3^)	AR ^4^ (°)	HR ^5^	CI ^6^ (%)	D50 ^7^ (μm)
0	0.27 ± 0.02	0.51 ± 0.03	43.9 ± 4.99	1.88 ± 0.02	46.9 ± 0.65	4.18 ± 0.03
5	0.33 ± 0.01	0.55 ± 0.04	40.3 ± 4.59	1.67 ± 0.14	40.0 ± 5.23	11.2 ± 0.12
10	0.52 ± 0.01	0.67 ± 0.00	39.5 ± 3.98	1.29 ± 0.03	22.7 ± 1.76	44.2 ± 0.17
20	0.51 ± 0.02	0.71 ± 0.01	36.3 ± 4.54	1.39 ± 0.05	27.8 ± 2.79	32.5 ± 0.21
30	0.61 ± 0.00	0.76 ± 0.00	27.9 ± 4.16	1.26 ± 0.00	20.4 ± 0.14	72.7 ± 2.99

^1^ Weight parentage of polyvinylpyrrolidone/vinyl acetate copolymer in the granule, ^2^ bulk density, ^3^ tapped density, ^4^ angle of repose, ^5^ Hausner ratio, ^6^ Carr index, ^7^ mass median diameter.

**Table 3:** Measured and calculated glass transition temperatures of SGR granules.

PVP-VA (%)	*T*_g expt_^1^ (°C)	*T*_g calc_^2^ (°C)	Δ*T*_g_^3^ (°C)
0	215.4	n/a	n/a
5	206.9	202.0	4.9
10	203.9	194.3	9.6
20	191.7	175.0	16.7
30	178.7	157.5	21.2

^1^*T*_g_ experimental, ^2^ *T*_g_ calculated, ^3^ *T*_g_ experimental − *T*_g_ calculated.
